# Effect of *Brucella* effector protein BtpB regulating apoptosis by targeting host protein YWHAZ

**DOI:** 10.1186/s13567-026-01840-9

**Published:** 2026-09-24

**Authors:** Lei Zhang, Yuxin Lv, Jingjing Chen, Yueting Wang, Yumeng Zhou, Yuxin Sun, Jingbo Gao, Yuqi Wang, Huan Zhang, Zeliang Chen

**Affiliations:** 1 Key Laboratory of Livestock Infectious Diseases, Ministry of Education, College of Animal Science and Veterinary Medicine, Shenyang Agricultural University, Shenyang, China; 2 Institute of Veterinary Medicine, Xinjiang Academy of Animal Science, Urumqi, China

**Keywords:** *Brucella*, BtpB, YWHAZ, apoptosis, protein interaction

## Abstract

*Brucella* is a successful intracellular bacteria, regulating various physiological activities within host cells by secreting multiple virulence factors. However, the specific molecular mechanisms underlying these processes remain unclear. Through interactions with host proteins, *Brucella* can influence cellular physiological processes to evade immune responses and enhance the bacteria survival and reproductive abilities. The type IV secretion system (T4SS) of *Brucella* plays a significant role in the process of *Brucella* infecting cells. This study revealed the interaction between BtpB and YWHAZ (14–3-3ζ) by analyzing the interaction network of 112 host proteins interacting with *Brucella* effector protein BtpB. Furthermore, we discovered that BtpB regulates the expression of YWHAZ protein and the deletion of its TIR domain abolishes this interaction as well as the effect on YWHAZ protein expression. Moreover, BtpB activates pro-apoptotic markers leading to apoptosis induction in cells. Our results provide mechanistic evidence that BtpB-dependent apoptosis regulation is contingent upon interaction with YWHAZ, and further characterize YWHAZ as a host factor governing *Brucella*'s intracellular survival capacity. This study provides valuable understanding of the immune response mechanism against intracellular bacterial infections in hosts and offers a theoretical basis for future prevention and treatment strategies for Brucellosis.

**Supplementary Information:**

The online version contains supplementary material available at https://doi.org/10.1186/s13567-026-01840-9.

## Introduction

*Brucella* is an intracellular facultative parasite capable of successful survival and replication within macrophages during cellular infection [1, 2]. These Gram-negative bacteria infect a diverse array of land and aquatic mammals, including swine, cattle, goat, sheep, dogs, dolphins, whales, seals, and desert wood rats [3]. The *Brucella* demonstrates adaptability to low pH, nearly anaerobic environments, and limited nutrient availability, consequently, it exhibits prolonged survival in diverse habitats including water, dust, soil, feces, meat, dairy products, and aborted fetuses [4, 5]. Brucellosis in humans typically presents with high, undulating fever. However, chronic brucellosis may affect many host organs, leading to arthritis, orchitis, hepatitis, encephalomyelitis and endocarditis [6, 7]. Arthritis is the predominant clinical complication of brucellosis [8]. The disease's polymorphic clinical presentation often complicates diagnostic procedures. Despite over a century of eradication efforts, *Brucella* infections persist even in developed nations, with no licensed human vaccine currently available [9]. *Brucella* species are classified as Category B bioterrorism agents due to their low infectious dose and potential for airborne transmission [10]. Moreover, *Brucella* possesses the ability to modulate host immune responses and displays a pronounced tropism for specific tissues such as fetal lungs [11].

The type IV secretion system (T4SS) is an important virulence factor of *Brucella* and is composed of 12 protein complexes encoded by the VirB operon. T4SS exerts its function through its secreted 16 effector proteins [12]. The effector proteins target key signaling pathways in host cells, inducing host immune responses while promoting the survival and replication of *Brucella* within host cells to facilitate persistent infection. In *Brucella* spp., they identified a TIR-containing protein, designated BtpB, which is translocated into host cells, interferes with dendritic cell activation, and disrupts toll-like receptor signaling to suppress innate immune responses [13]. Recent investigations have revealed that TIR domains of BtpA and BtpB regulate host cell energy metabolism through niacinamide adenine dinucleotide hydrolysis 1 (NAD1) [14]. The specific target protein of BtpB within host cells and its impact on *Brucella* intracellular survival remain elusive. Therefore, providing a comprehensive and thorough elucidation of protein function holds immense significance in unraveling the precise pathogenic mechanism of *Brucella*.

Cell death by apoptosis is a common response of mammalian cells to a wide variety of bacterial infections [15]. The virulence factors unique to *Brucella*, such as lipopolysaccharide (LPS), outer membrane proteins (OMPs), T4SS, and their effector proteins, have different effects on cellular apoptosis. The OMP31 protein derived from *Brucella* melitensis 16M has been reported to inhibit macrophage apoptosis by triggering tumor necrosis factor (TNF)-α [16] and Omp25 protein can inhibit apoptosis in BV2 microglial cells [17]. Previous studies have shown that the absence of T4SS effectors VceC and VceA can impact *Brucella*'s regulation of cellular apoptosis [18]. Deletion of VceA inhibits apoptosis in *Brucella*-infected human trophoblast cells and VceC inhibits ER stress-induced apoptosis in the CHOP pathway of goat trophoblast cells [19, 20]. In our previous studies, *Brucella* effector protein BspF inhibit host cell apoptosis through the crotonylation of p53 [21–23]. Furthermore, it has been discovered that the nuclear effector protein BspJ of *Brucella* can inhibit the apoptosis of macrophages [24]. Existing studies have revealed that the *Brucella* T4SS effector protein BtpB can promote RAW264.7 cell apoptosis [25], though the underlying mechanism remains unclear.

In this study, we identified 112 host cell proteins that interact with the *Brucella* effector protein BtpB through interaction omics analysis, including the host apoptosis-associated protein YWHAZ (14–3-3ζ). This study confirmed the BtpB – YWHAZ interaction and demonstrated that BtpB modulates YWHAZ protein expression in a manner dependent on its TIR domain. Moreover, our data indicate that BtpB exerts a modest pro-apoptotic effect, which is linked to its interaction with YWHAZ. These findings offer insights into the host immune response to intracellular bacterial infection and provide a conceptual framework for future strategies aimed at disease prevention and treatment.

## Materials and methods

### Strains, cells, plasmid and reagents

The wild-type strain *Brucella* abortus 2308 and the mutant strain *Brucella* 2308 Δ*btpB* used in this study were kindly provided by the Academy of Military Science. The eukaryotic expression vectors pCMV-HA and pCMV-Tag2B used in this experiment were archived in our laboratory. The eukaryotic expression plasmids pCMV-HA-BtpB and pCMV-HA-BtpBΔTIR were constructed in this study, and the plasmid pCMV-Tag2B-YWHAZ was archived in our laboratory. The HEK-293T and HeLa cell lines were also archived in our laboratory. The primers for quantitative PCR of apoptosis-related genes AIF, Bcl-2, Bax and Caspase 3 were designed and synthesized by Shengong Biotechnology (Shanghai) Co., Ltd.

### Eukaryotic plasmid transfection

The eukaryotic expression plasmids pCMV-HA-BtpB, pCMV-HA-BtpBΔTIR, and pCMV-Tag2B-YWHAZ were separately transfected into HEK-293T cells. Cells were harvested for protein extraction 30 h after transfection. All transfections were carried out in a laminar flow cabinet according to the following procedure: HEK-293T cells in good condition were plated into 10 cm^2^ culture dishes and incubated overnight at 37 ℃ under 5% CO_2_. Transfection was performed when cells attained 70–80% confluence (monolayer), as verified by microscopic examination. Transfection complexes were prepared according to the manufacturer’s protocol for Lipofectamine 2000: plasmid and Lipofectamine 2000 solutions were prepared separately in serum-free DMEM and incubated at room temperature for 5 min after gentle mixing. The lipid-DMEM mixture was then slowly combined with the plasmid-DMEM solution, gently mixed again, and incubated at room temperature for 20 min. The resulting complex was added dropwise to the cells (after removing an equal volume of the original medium), followed by incubation at 37 ℃ with 5% CO_2_. After a 6-h incubation period, the cultures were replenished with maintenance medium (2% FBS), and the cells were collected at 30 h post-transfection.

### Coomassie staining and silver staining

The electrophoresed PAGE gel was placed in a glass dish containing 200 mL of Coomassie brilliant blue staining solution and stained on a shaking platform for 12 h to ensure complete staining. Subsequently, the gel was transferred to another glass dish containing 200 mL of destaining solution and destained with visual inspection every 30 min until the background was clear. Finally, the gel was imaged and documented using a ChemiDoc XRS digital gel imaging and analysis system.

With greater sensitivity than Coomassie staining, silver staining allows for the identification of low‑abundance proteins. The PAGE gel was transferred to a glass or enamel tray and rinsed twice with deionized water for 2 min each time. The gel was then immersed in silver staining solution until fully covered and stained at room temperature for 10 min with gentle shaking on a platform to ensure even coloration. After discarding the staining solution, the gel was quickly rinsed three times with deionized water for 30 s each time. The gel was transferred to developing solution and incubated at room temperature with gentle shaking until protein bands became clearly visible. The gel was then placed in stop solution for 1 min to terminate the developing reaction. Finally, the silver‑stained PAGE gel was photographed for documentation.

### Co-IP and WB

Western blotting was performed according to established methods. Briefly, HEK-293T cells transfected with the eukaryotic plasmids using the protocol described above were harvested 24 h post-transfection. After being washed with phosphate-buffered saline (PBS), the cells were subjected to lysis in ice-cold buffer supplemented with a protease inhibitor cocktail (Sigma-Aldrich). The lysate was incubated on ice for 1 h in a buffer containing 1% (v/v) Triton X-100, 150 mM NaCl, 25 mM Tris–HCl (pH 7.4), and 0.2% sodium azide. Subsequently, the lysate was centrifuged at 16 000 × *g* for 15 min at 4 ℃. Proteins were separated by 12% SDS-PAGE and electrophoretically transferred onto a nitrocellulose membrane at 200 mA for 30 min. Following a 1 h blocking step in TBST (Tris-buffered saline with 0.1% Tween-20) at 37 °C, the membrane was probed with the primary antibody overnight at 4 °C. After incubation, the membrane was washed three times with 1× TBST (5 min per wash) and then incubated with an appropriately diluted secondary antibody solution at room temperature for 2 h with gentle shaking. Following another three washes with 1× TBST (5 min per wash), the membrane was developed using a chemiluminescent substrate kit according to the manufacturer’s instructions. Signal detection and image acquisition were performed using a chemiluminescence imaging system. The molecular weight marker used is Beijing Solarbio Science & Technology Co., Ltd. (Catalog No.: PR1910).

### Quantitative real time‑PCR (qRT‑PCR)

According to the above method, Hela epithelial cells were infected with *Brucella* 2308 strain and *Brucella* 2308Δ*btpB* strains. Cells not infected with *Brucella* were used as a control. At 12 and 24 h post-infection, as described before, total RNA was extracted from the remaining homogenate using Trizol. To the remaining homogenate, 1mL of Trizol was added for total RNA extraction. DNase I treatment was applied to the RNA samples for the removal of contaminating genomic DNA. Reverse transcription of total RNA into cDNA was carried out with random hexamer primers and the SuperScript II kit (Invitrogen, Carlsbad, CA, USA) in accordance with the provided protocol. Apoptosis-related genes Bcl-2, Caspase 3, Bax, and AIF were selected for detection.

### Flow cytometry

A density of 5 × 10^5^ cells per well was used to seed healthy HeLa cells in 6-well culture plates. Plasmids were transfected into cells and samples were collected after 24 and 48 h for staining treatment. Cells in the 6-well plate were collected in a 1.5 mL centrifuge tube, centrifuged at 1000 × *g* for 5 min, and the cell culture medium was discarded. PBS was added for washing 1–2 times, centrifuged at 1000 × *g* for 5 min, and the PBS was discarded. Cell sediment was gently resuspended in 195 µL of Annexin V-FITC binding solution, then 5 µL of Annexin V-FITC was added and mixed well. Subsequently, 10 µL of propidium iodide staining solution was added and gently mixed. The mixture was left to stand at 20–25 °C for 10–20 min, after which it was chilled on ice. Light protection can be provided by using aluminum foil. Detection was performed within one hour.

### *Brucella* culture and cell infection assay

*Brucella* abortus strain S2308 was retrieved from storage at −80 °C and streaked onto Tryptic Soy Agar (TSA) plates. A single isolated colony was transferred into Tryptic Soy Broth (TSB) and grown at 37 °C with shaking at 180 rpm for 24 h, following a 2–3 day incubation period at 37 °C. Bacterial cells obtained from 1 mL of culture by centrifugation at 4500 × *g* for 5 min were washed twice with PBS after the supernatant was removed. Finally, the bacteria were resuspended in PBS to an appropriate density. The bacterial concentration was standardized using the McFarland turbidimetry method.

For cell infection assays, HeLa cells were seeded in 6-well plates and grown for 24 h to achieve a density of approximately 1.0 × 10^6^ cells per well. Cells were then inoculated with either the wild-type *Brucella* abortus 2308 or its isogenic mutant Δ*btpB* at a multiplicity of infection (MOI) of 200. After a 2 h incubation at 37 °C to allow for bacterial internalization, the monolayers were gently washed with PBS to remove non-adherent bacteria. Cells were incubated for 1 h in medium containing 50 µg/mL gentamicin to clear extracellular bacteria. Under a humidified atmosphere of 5% CO_2_ at 37 °C, the infection was sustained in fresh DMEM supplemented with 2% FBS and 25 µg/mL gentamicin.

### Statistical analysis

All Co-IP and western blot experiments were performed in three independent biological replicates. Quantification of protein bands was conducted using ImageJ software (version 1.8.0). To normalize for loading variations, the densitometric value of each target band was divided by that of the corresponding internal control. The resultant normalized values were subjected to univariate statistical analysis. Data are presented as the mean ± SD (or SEM) from three independent experiments. Statistical significance was defined as follows: *p < 0.05, **p < 0.01, ***p < 0.001, and ****p < 0.0001; differences with *p > 0.05 were considered not significant.

## Results

### Investigation of the interaction between *Brucella* effector protein BtpB and YWHAZ.

**Figure 1:**
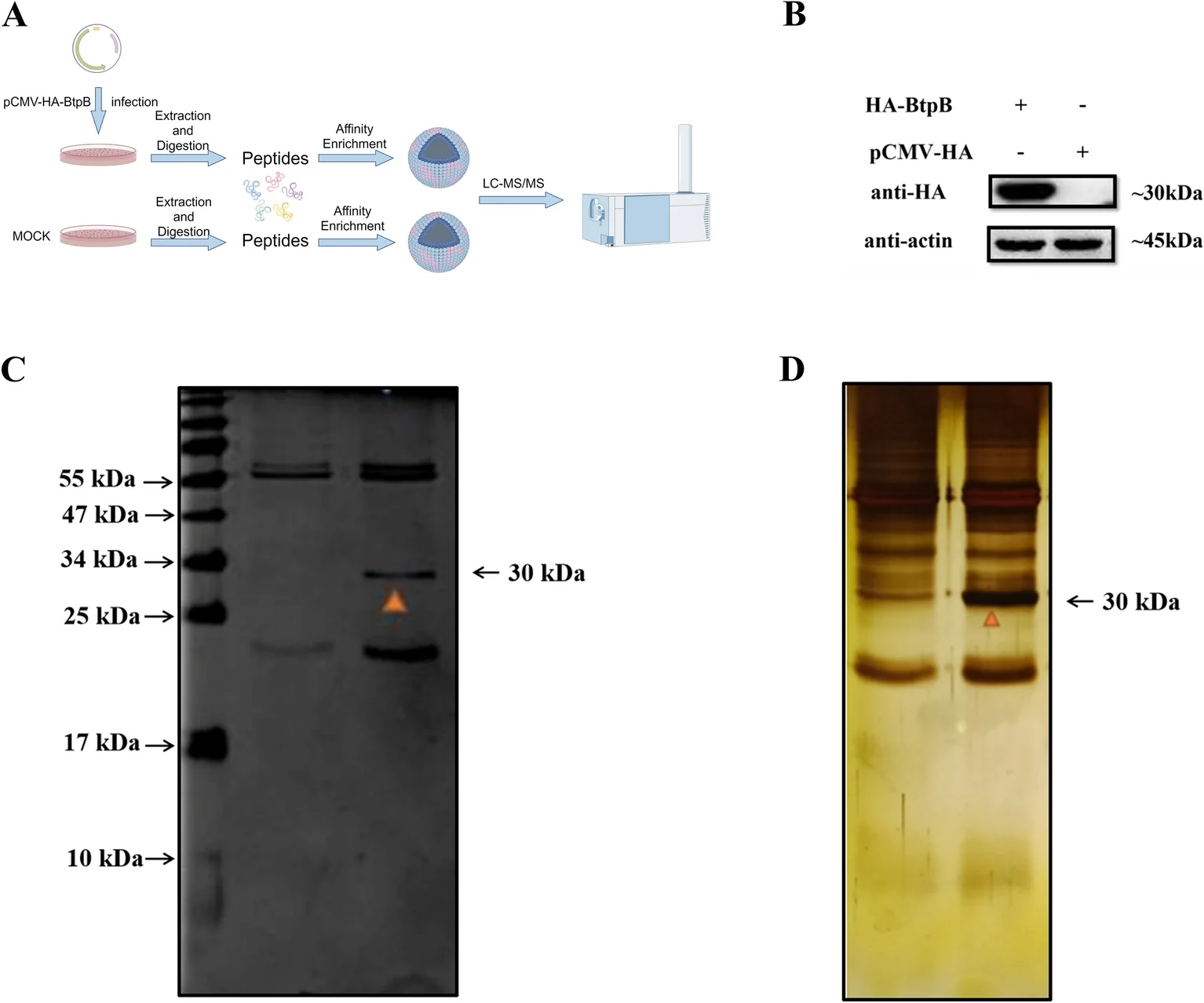
**LC–MS/MS analysis of Brucella effector protein BtpB.** **A** Host proteins interacting with the BtpB protein were identified by LC–MS/MS through interactomics. **B** The recombinant plasmid pCMV-HA-BtpB was transfected into HEK-293T cells and expressed for 30 h, after which the collected samples were analyzed by western blot. **C** HA-tagged BtpB was transfected into HEK-293T cells. Cells were harvested 30 h after transfection. The samples were subjected to SDS-PAGE electrophoresis followed by Coomassie staining. **D** HA-tagged BtpB was transfected into HEK 293 T cells. Cells were harvested 30 h after transfection. The samples were separated by SDS-PAGE and subsequently visualized by silver staining.

To identify host proteins that interact with BtpB, host-derived interacting partners of this bacterial effector protein were systematically screened for using an liquid chromatography-tandem mass spectrometry (LC-MS/MS) approach (Figure 1A). The BtpB gene was cloned into the eukaryotic expression vector pCMV-HA, and the resultant recombinant plasmid pCMV-HA-BtpB was transfected into host cells. Western blot analysis of harvested samples confirmed that the plasmid stably expressed a 30 kDa-specific protein corresponding to BtpB (Figure 1B). The recombinant plasmid pCMV-HA-BtpB was transfected into HEK-293T cells, which were then cultured for 30 h to allow protein expression. After harvesting the cells, the samples followed by sodium dodecyl sulfate–polyacrylamide gel electrophoresis (SDS-PAGE) separation. Subsequent Coomassie brilliant blue staining and silver staining both detected a distinct protein band at an approximate molecular weight of 30 kDa, consistent with the expected size of BtpB (Figure 1C and D). The gel band containing the target protein was excised and subjected to LC–MS/MS analysis. The results indicated that a total of 112 host proteins interacted with BtpB (Refer to Additional file 1 for details).

**Figure 2:**
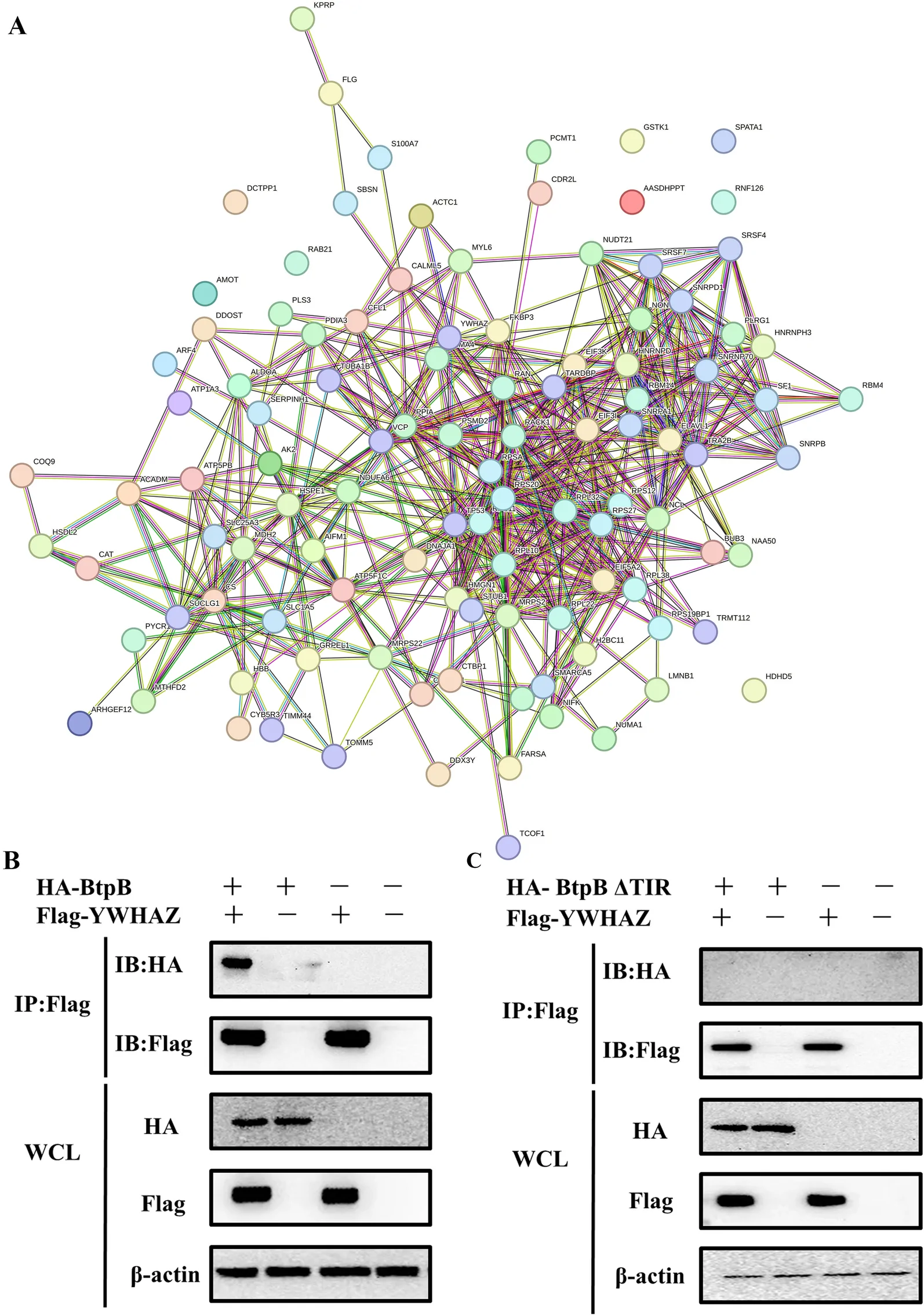
**Identification of host proteins interacting with BtpB and validation by Co-IP.** **A** Interaction networks of BtpB interacting host proteins. Network analysis of the host proteins that interacted with BtpB was performed using Proteome Discoverer 2.5. The mass spectrometry data identified a specific interaction between BtpB and YWHAZ. **B** Verification of the protein–protein interaction by Co-IP assay. HA-tagged BtpB and Flag-tagged YWHAZ were co-transfected into HEK 293 T cells. Cells were harvested 30 h after transfection for Co-IP assay. **C** HA-tagged BtpBΔTIR and Flag-tagged YWHAZ were co-transfected into HEK 293 T cells and then processed as in (A).

Interaction network analysis was performed on the 112 host proteins interacting with BtpB using Proteome Discoverer 2.5 software, revealing that these proteins belong to the same interaction network (Figure 2A). The proteins depicted in interaction network diagrams primarily participate in diverse biological processes, encompassing protein modification, cellular apoptosis, cellular immunity, gene expression regulation, transcriptional control, translational machinery operation, and intracellular cargo transportation. Among them, YWHAZ, a member of the 14-3-3 protein family, functions as a pivotal hub protein involved in multiple signal transduction pathways and plays a crucial role in regulating apoptosis and interacts with various proteins within the apoptotic pathway to exert its effects [26].

The interaction between BtpB and YWHAZ was initially identified by mass spectrometry. This interaction was subsequently validated by co-immunoprecipitation (Co-IP) assays and further corroborated by western blot analysis in HEK-293T cells, confirming the association between the *Brucella* effector BtpB and the host protein YWHAZ (Figure 2B). The TIR domain, a critical structural module of BtpB and also found in BtpA, mimics MAL/TIRAP and competes with TLR downstream signaling molecules, thereby suppressing NF-κB activation and dampening inflammatory responses [27]. To investigate whether the TIR domain is required for the interaction between BtpB and YWHAZ, pCMV-HA-BtpBΔTIR plasmid was constructed. HEK-293T cells were transfected with both pCMV-HA-BtpBΔTIR and pCMV-Tag2B-YWHAZ plasmids. The results demonstrated that BtpBΔTIR failed to interact with YWHAZ (Figure 2C). These findings indicate that BtpB interacts with YWHAZ in a TIR domain-dependent manner.

### BtpB reduces YWHAZ expression through TIR domain.

**Figure 3:**
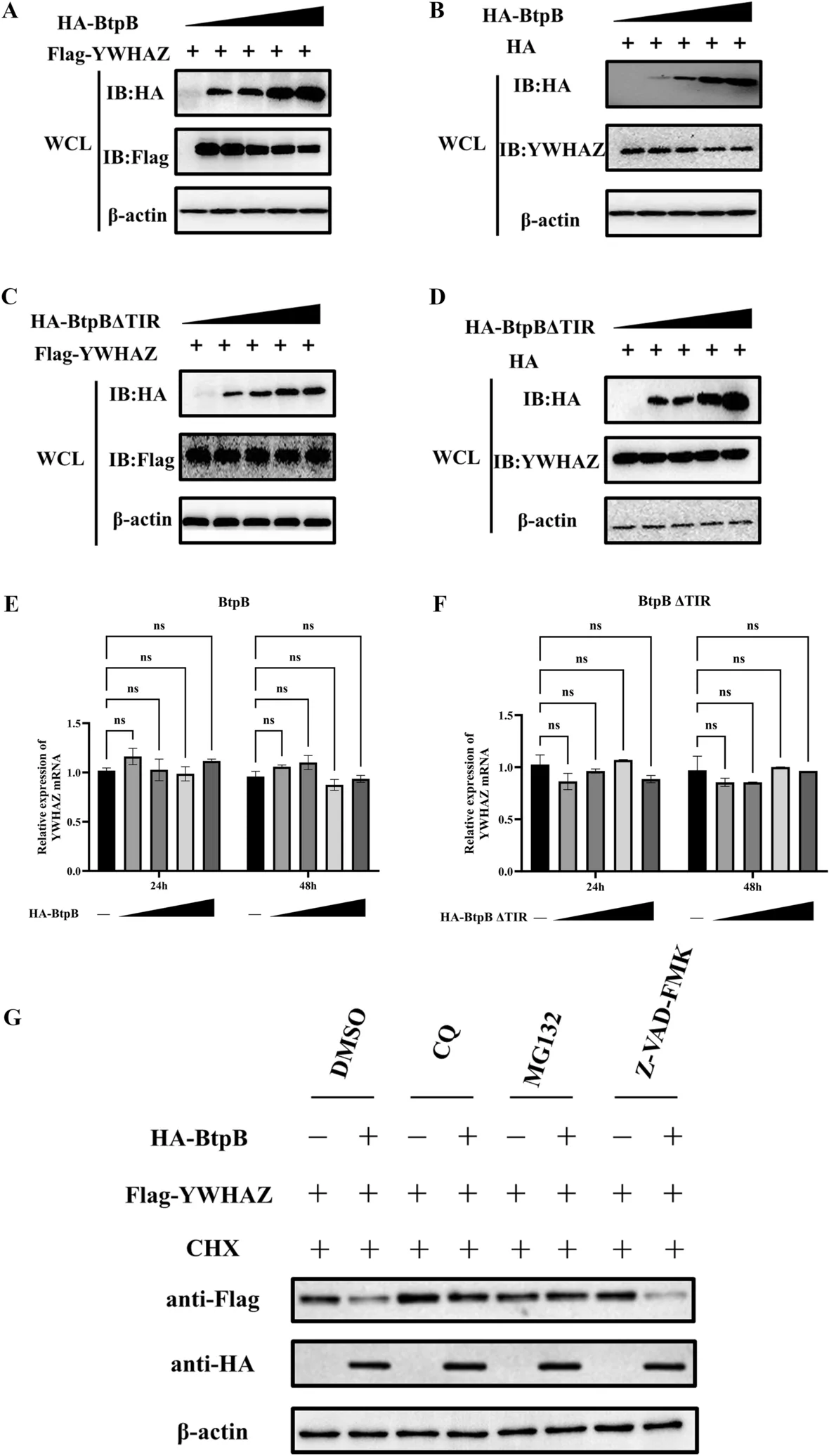
**BtpB inhibits YWHAZ protein expression through TIR domain.** **A** HEK-293T cells were co-transfected with increasing doses of HA-tagged BtpB and Flag-tagged YWHAZ. Cell harvesting was performed 30 h post-transfection, followed by western blot analysis. **B** The effect of increasing BtpB transfection dose on YWHAZ protein expression was detected by western blotting by using anti-YWHAZ and anti-HA antibodies. **C** Western blotting was used to detect the effect of increasing BtpBΔTIR transfection dose on the expression of YWHAZ protein by using anti-HA and anti-Flag antibodies. **D** The effects of increasing BtpBΔTIR transfection dose on YWHAZ protein expression was detected by western blotting by using anti-HA and anti-YWHAZ antibodies. **E** HEK-293T cells were transfected with increasing doses of HA-tagged BtpB. Total RNA was isolated from the cells at 24 h and 48 h post-transfection and reverse transcribed into cDNA, and the mRNA transcription levels of YWHAZ were detected by qRT-PCR. **F** HeLa cells transfected with increasing doses of HA-tagged BtpBΔTIR were harvested at 24 h and 48 h for RNA isolation, reverse transcription, and qRT-PCR analysis of YWHAZ mRNA. **G** HEK-293T cells transfected with HA-BtpB and Flag-YWHAZ plasmids were treated with DMSO, CQ, MG132, or Z-VAD-FMK, and then analyzed by western blotting.

We further investigated whether BtpB could influence the expression of YWHAZ following the observed interaction between these two proteins. The protein expression levels of YWHAZ, including both exogenous and endogenous forms, were evaluated using western blot analysis. The results revealed an inverse correlation between BtpB protein levels and YWHAZ expression, showing that YWHAZ protein levels progressively decreased with increasing BtpB expression (Figure 3A and B). In contrast, when the expression levels of BtpBΔTIR protein was elevated, no significant changes were observed in either exogenous or endogenous YWHAZ protein levels (Figure 3C and D). To determine whether YWHAZ mRNA levels are affected by BtpB expression, YWHAZ transcript levels were measured by qRT-PCR after BtpB expression in cells. The results showed that BtpB expression did not significantly alter YWHAZ mRNA levels compared with the control group, indicating that the reduction in YWHAZ protein is not due to transcriptional inhibition (Figure 3E and F). To further investigate the degradation pathways involved in YWHAZ degradation triggered by BtpB, BtpB-expressing cells were treated with the proteasomal inhibitor MG132, the autophagic-lysosomal pathway inhibitor chloroquine (CQ), and the pan-caspase inhibitor Z-VAD-FMK. Western blot analysis revealed that treatment with either MG132 or CQ significantly restored YWHAZ protein levels reduced by BtpB (Figure 3G). These findings suggest that BtpB downregulates intracellular YWHAZ protein expression through its TIR domain. The reduction in protein levels was shown not to be caused by transcriptional inhibition, but rather to occur via the proteasomal and autophagolysosomal pathways.

### *Brucella *2308 Δ*btpB* Deletion Strains Inhibit Host Cell Apoptosis

**Figure 4:**
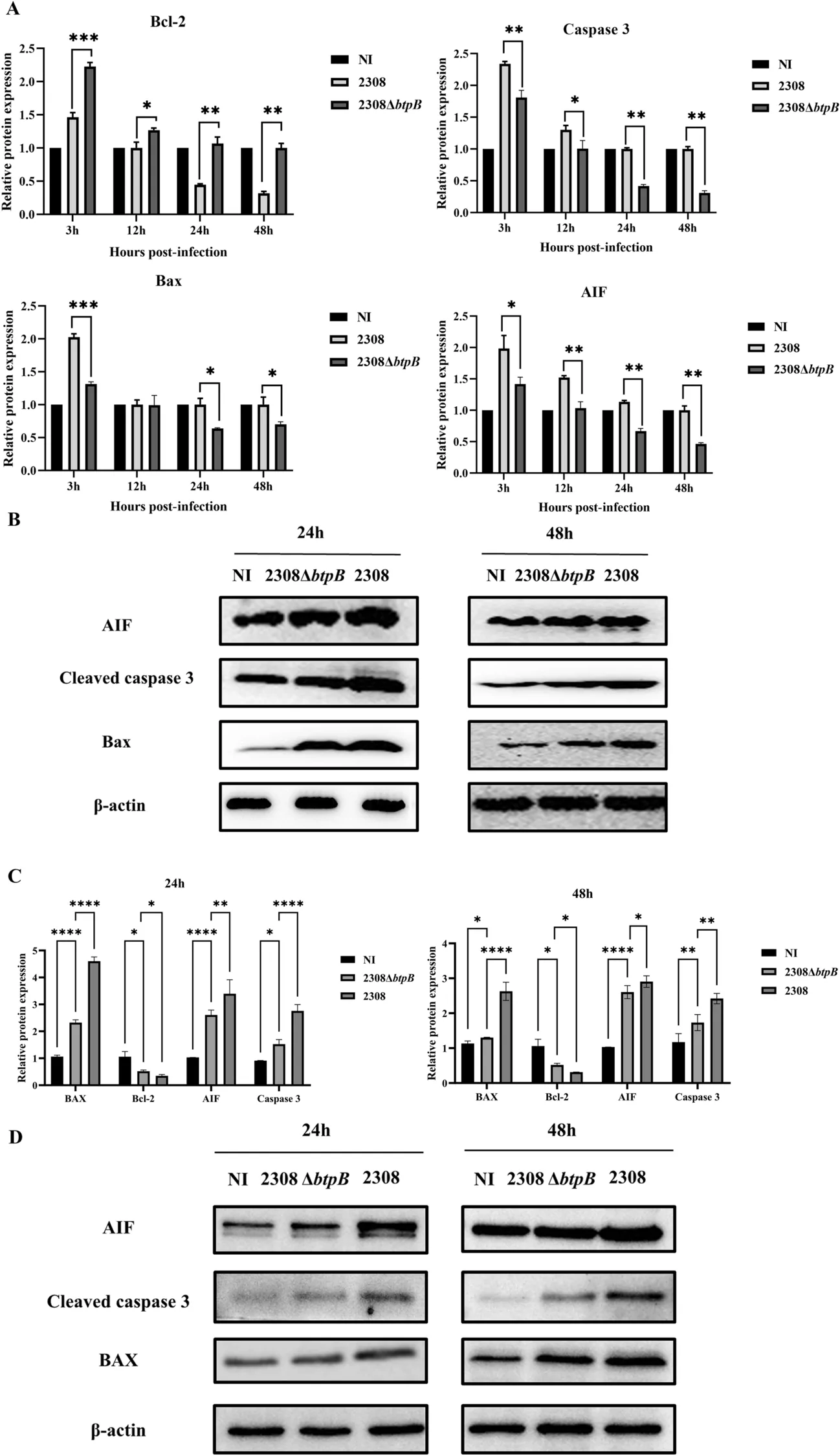
**The Brucella ∆btpB strain downregulated the transcription and protein expression of apoptosis-related genes and suppressed the cell apoptosis process.** **A** Quantification of transcriptional changes in apoptosis-associated genes*.* Hela cells were infected with 100 MOI of *Brucella* 2308 strain and *Brucella* 2308 Δ*btpB* strain. Total RNA was isolated from the cells at 3 h, 12 h, 24 h and 48 h post-infection and reverse transcribed into cDNA, and the mRNA transfection levels of apoptosis genes Bcl-2, Bax, Caspase 3 and AIF were detected by qRT-PCR. **B** Immunoblotting of AIF, Caspase 3, and Bax proteins in HeLa cells not infected or infected with different *Brucella* 2308 strains. Hela cells were infected with 100 MOI of *Brucella* 2308 strain and *Brucella* 2308 Δ*btpB* strain, and the none infected cells was used as a negative control, followed by analysis and detection of pro-apoptotic proteins AIF, Cleaved caspase 3 and Bax expression by western blot analysis. **C** RAW264.7 cells were infected with 100 MOI of *Brucella* 2308 and *Brucella* 2308 Δ*btpB* strain. Total RNA was isolated at 24 and 48 h post-infection, reverse transcribed, and the mRNA levels of apoptosis-related genes (Bcl-2, Bax, Caspase 3, and AIF) were measured by qRT-PCR. **D** Expression of the pro-apoptotic proteins AIF, Cleaved caspase 3, and Bax was detected by western blot analysis in RAW264.7 cells infected with 100 MOI of *Brucella* 2308 strain and *Brucella* 2308 Δ*btpB* strain.

YWHAZ, a member of the 14-3-3 protein family, is a central hub protein involved in numerous signal transduction pathways and plays a critical role in apoptosis [28]. Based on the interaction between BtpB and apoptosis-related protein YWHAZ, it is postulated that BtpB exerts a certain influence on host cell apoptosis. Bcl-2, Bax, Caspase 3 and AIF are crucial targets in the apoptosis pathway, and their expression levels undergo significant changes during the process of apoptosis, making them reliable indicators for detecting apoptosis [29]. To investigate the impact of BtpB on cellular apoptosis, HeLa cells were infected with *Brucella* 2308 strain and *Brucella* 2308 Δ*btpB* strain. Subsequently, total cellular RNA was extracted and qRT-PCR (quantitative Real-Time PCR) experiments were conducted to detect the transcription of genes associated with apoptosis. As shown in Figure 4A, *Brucella* infection induced a time-dependent decrease in the transcription of the anti-apoptotic gene Bcl-2 compared with non-infected cells. However, compared with *Brucella* 2308 strain infected cells, the transcription levels of the Bcl-2 gene was upregulated in *Brucella* 2308 Δ*btpB* strain infected cells. Conversely, the relative transcription levels of the Caspase 3, Bax, and AIF were significantly lower in cells infected with the *Brucella* 2308 Δ*btpB* strain compared with those infected with the *Brucella* 2308 strain. Collectively, these results indicate that BtpB deficiency promotes expression of the anti-apoptotic gene Bcl-2 and suppresses expression of pro-apoptotic genes during *Brucella* infection, supporting a role for BtpB in promoting host-cell apoptosis (Figure 4A). The protein expression levels of apoptosis-related proteins AIF, Caspase 3, and Bax were further detected by western blotting in cells infected with *Brucella* 2308 strain and *Brucella* 2308 Δ*btpB* strain (Figure 4B). The results demonstrated that the expression levels of AIF, Caspase 3, and Bax proteins decreased in both the *Brucella* 2308 and 2308 Δ*btpB* strain infected cells when compared with the none infected cells. Moreover, compared with the *Brucella* 2308 strain infected cells, the expression levels of AIF, Caspase 3, and Bax proteins were significantly reduced in the *Brucella* 2308 Δ*btpB* strain infected cells, indicating *Brucella* effector protein BtpB promotes apoptosis. The mRNA and protein levels of apoptosis-related genes were also examined in RAW264.7 cells infected with *Brucella* 2308 or the 2308 Δ*btpB* strain. Compared with the 2308 strain, the 2308 Δ*btpB* strain exhibited a weakened ability to induce apoptosis (Figure 4C and D). These results were consistent with those previously observed in HeLa cells. This indirectly implies a potential role of the effector protein BtpB in promoting apoptotic processes.

### BtpB affects the transcription and protein expression of apoptosis-related genes through its TIR domain.

Previous findings prompted us to hypothesize that BtpB-mediated regulation of YWHAZ may perturb apoptotic processes. To determine whether the pro-apoptotic function of BtpB depends on its interaction with YWHAZ, this interaction was disrupted by deletion of the TIR domain.

**Figure 5:**
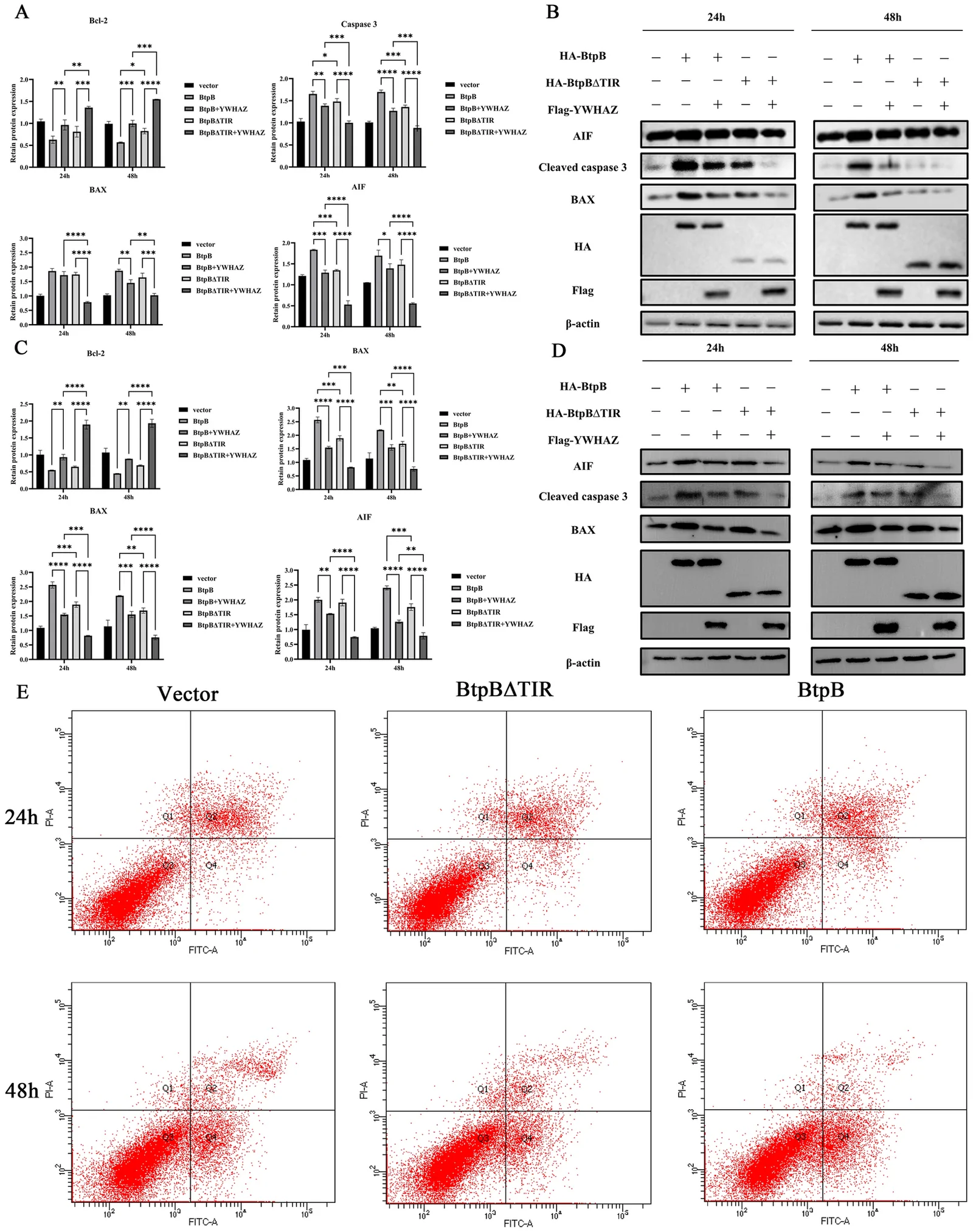
**BtpB affects the expression of genes and proteins related to apoptosis during infection.** The data shown are representative of three independent experiments. **A** The transcription levels of apoptosis-associated genes. Hela cells were co-transfection with pCMV-HA-BtpB, pCMV-HA-BtpB∆TIR and pCMV-Tag2B-YWHAZ plasmids. RNA was isolated from the cells at 24 h and 48 h post-transcription. **B** Western blot analysis of apoptosis-related proteins. pCMV-HA-BtpB, pCMV-HA-BtpB∆TIR and pCMV-Tag2B-YWHAZ plasmids were transfected into Hela cells. Protein samples were collected 24 and 48 h after transfection and detection of pro-apoptotic proteins AIF, Caspase 3 and Bax expression by western blot. **C** The transcription levels of apoptosis-associated genes. RAW264.7 cells were co-transfection with pCMV-HA-BtpB, pCMV-HA-BtpB∆TIR and pCMV-Tag2B-YWHAZ plasmids. RNA was isolated from the cells at 24 h and 48 h post-transcription. **D** Western blot analysis of apoptosis-related proteins. pCMV-HA-BtpB, pCMV-HA-BtpB∆TIR and pCMV-Tag2B-YWHAZ plasmids were transfected into RAW264.7 cells. Protein samples were collected 24 and 48 h after transfection and detection of pro-apoptotic proteins AIF, Caspase 3 and Bax expression by western blot. **E** Flow cytometry was employed to assess the apoptotic rate of different groups at the cellular levels. Hela cells were transfected with pCMV-HA, pCMV-HA-BtpB and pCMV-HA-BtpBΔTIR plasmids for 24 and 48 h. The cells were stained with Annexin V-FITC and PI and then detected by flow cytometry. Q1: PI is positive and Anenexin V is negative cells; Q2: Both were negative cells; Q3: Both are positive cells; Q4: Anenexin V is a positive cell while PI is negative.

To investigate whether BtpB modulates the mRNA transcriptional levels and the protein expression levels of apoptosis-related genes through its TIR domain, HeLa cells and RAW264.7 cells were transfected with either pCMV-HA-BtpB or pCMV-HA-BtpBΔTIR plasmids, and we analyzed the mRNA transcriptional levels and the protein expression levels of apoptosis-related markers at 24 and 48 h post-transfection. Both BtpB and BtpBΔTIR plasmids transfection led to decreased transcription of the anti-apoptotic gene Bcl-2, while the transcript levels and the protein expression levels of pro-apoptotic genes Caspase 3, Bax, and AIF were significantly upregulated, while the absence of the TIR domain partially diminished this enhancing effect. Furthermore, to investigate the effect of YWHAZ on BtpB-induced apoptosis, YWHAZ was co-transfected with either BtpB or BtpBΔTIR plasmids into HeLa and RAW264.7 cells,and the transcription levels and the protein expression levels of apoptosis-related genes were examined at 24 and 48 h post-expression (Figure 5A and D). The results showed that the BtpB-induced upregulation of AIF, Caspase 3, and Bax at both the transcription levels and the protein levels were partially reduced by YWHAZ expression.

Previous experiments demonstrated that *Brucella* 2308 Δ*btpB* strain possesses the ability to inhibit apoptosis, suggesting a potential pro-apoptotic role for BtpB. This hypothesis was subsequently verified by flow cytometry analysis. Transfection of Hela cells with pCMV-HA, pCMV-HA-BtpB, and pCMV-HA-BtpBΔTIR plasmids revealed that, as shown in Figure 5E, both pCMV-HA-BtpB, and pCMV-HA-BtpBΔTIR plasmids transfected cells exhibited time-dependent increases in apoptotic rates compared with the empty vector transfected cells. Notably, the apoptotic rate in the pCMV-HA-BtpBΔTIR plasmids transfected cells were consistently lower than that in the pCMV-HA-BtpB plasmids transfected cells. These results confirmed that BtpB-induced apoptosis was closely associated with both its TIR domain and its interaction with YWHAZ, as shown in Tables 1 and 2.

**Table 1:** **The apoptosis statistic of plasmids pCMV-HA-BtpB and pCMV-HA-BtpB∆TIR transfected HeLa cells for 24 h by FCM assay**

Group	Normal cells	The early apoptotic cells (%)	The late apoptotic cells (%)
Vector	81.70 ± 0.39	11.46 ± 0.25	4.10 ± 0.56
pCMV-HA-BtpB	82.40 ± 0.54	13.70 ± 0.38	5.50 ± 0.37
pCMV-HA-BtpB∆TIR	81.40 ± 0.26	12.40 ± 0.36	4.91 ± 0.45

**Table 2:** **The apoptosis statistic of plasmids pCMV-HA-BtpB and pCMV-HA-BtpB∆TIR transfected HeLa cells for 48 h by FCM assay**

Group	Normal cells	The early apoptotic cells (%)	The late apoptotic cells (%)
Vector	75.70 ± 0.39	4.80 ± 0.27	13.10 ± 0.48
pCMV-HA-BtpB	78.80 ± 0.26	6.10 ± 0.31	17.90 ± 0.36
pCMV-HA-BtpB∆TIR	76.60 ± 0.34	5.45 ± 0.33	15.27 ± 0.49

### YWHAZ restricts the intracellular survival of *Brucella*

**Figure 6:**
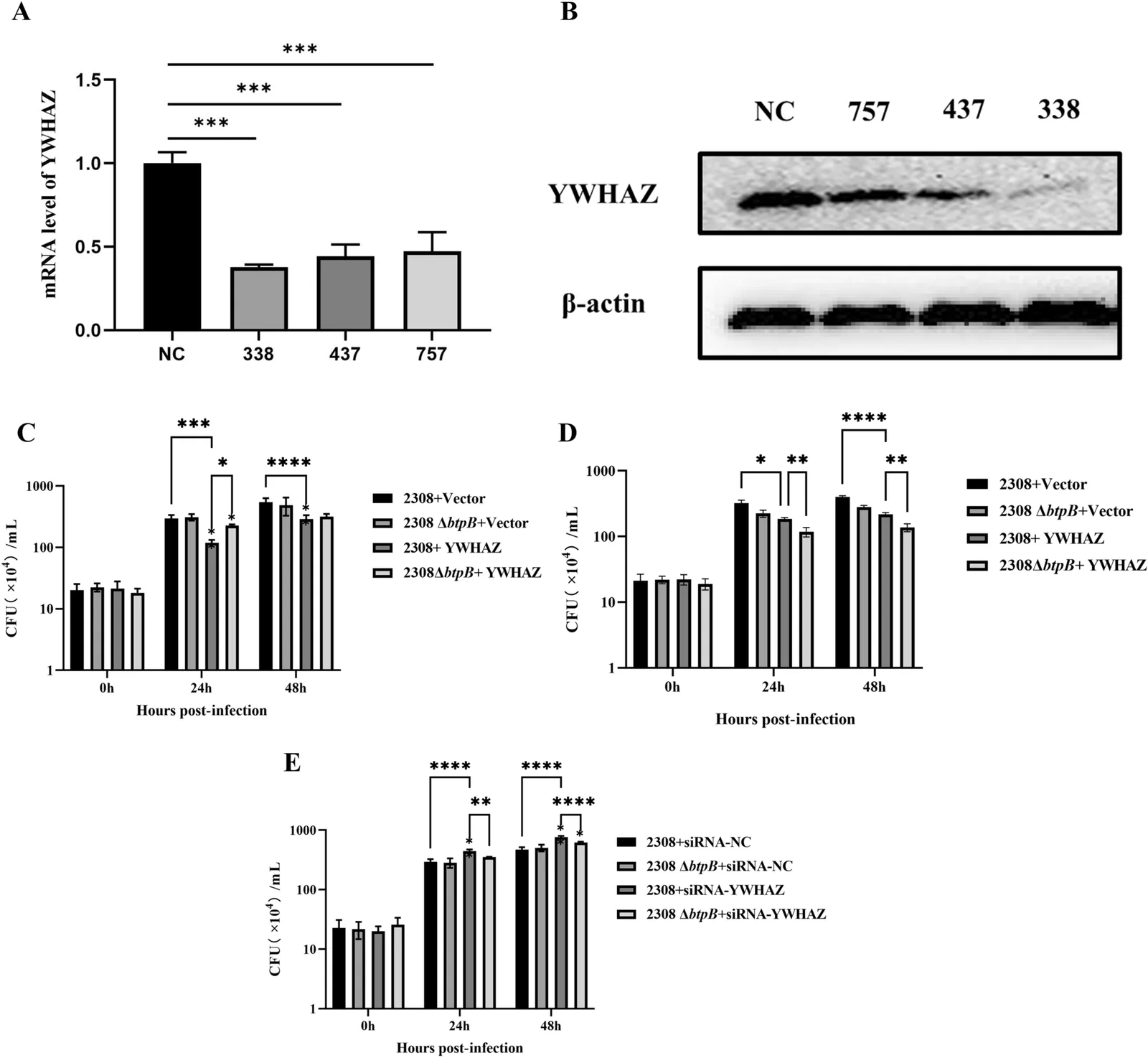
**YWHAZ inhibited Brucella intracellular survival.** **A** Effects of siRNAs on transcription levels of YWHAZ. **B** Effects of siRNAs on protein expression of YWHAZ. **C** Effect of YWHAZ overexpression on the intracellular survival of *Brucella* 2308 strain and *Brucella* 2308Δ*btpB* strain in HeLa cells. **D** Effect of YWHAZ overexpression on the intracellular survival of *Brucella* 2308 strain and *Brucella* 2308Δ*btpB* strain in RAW264.7 cells. **E** Effect of silencing YWHAZ on intracellular survival of *Brucella* 2308 strain and *Brucella* 2308Δ*btpB* strain*.* * *p* < 0.05, ** *p* < 0.01.

The role of YWHAZ in the intracellular survival of *Brucella* remains unclear. To investigate the impact of this host protein on the intracellular survival of *Brucella* 2308 strain, the expression of YWHAZ in HeLa cells was either overexpressed or silenced. Three siRNA targeting sequences for the YWHAZ gene were designed, and validation experiments were performed to evaluate the knockdown efficiency of each siRNA. As shown in Figure 6A, siRNA-338 demonstrated significant inhibition of YWHAZ mRNA expression. This inhibitory effect on YWHAZ protein levels was further confirmed by immunoblotting (Figure 6B). Consequently, siRNA-338 was employed in all subsequent experiments. Overexpression of YWHAZ was found to significantly inhibit the intracellular survival of *Brucella* 2308 strain, whereas this inhibitory effect was markedly attenuated in the *Brucella* 2308 Δ*btpB* strain. Notably, this inhibition was observed in both HeLa cells and RAW264.7 cells. However, in YWHAZ-overexpressing RAW264.7 cells, replication of the 2308 Δ*btpB* strain was more strongly inhibited compared with that of the 2308 strain, which shows a result that differed from what was observed in HeLa cells (Figure 6C and D). Furthermore, silencing of YWHAZ significantly enhanced the intracellular survival of *Brucella* 2308 strain. However, the survival advantage conferred by YWHAZ knockdown was substantially attenuated in the *Brucella* 2308 Δ*btpB* strain (Figure 6E). The results demonstrate that YWHAZ overexpression reduces the intracellular abundance of live *Brucella* and impairs their survival ability. Conversely, the absence of YWHAZ enhances bacterial load within cells and promotes *Brucella*'s intracellular survival. Moreover, as the duration of bacterial infection increases, compared with the *Brucella* 2308 Δ*btpB* strain, YWHAZ overexpression exerts a more pronounced inhibitory effect on intracellular survival of the *Brucella* 2308 strain, while silencing YWHAZ exhibits a more pronounced promoting effect on its intracellular survival. We speculate that the difference in intracellular survival between the two strains may be related to the interaction between BtpB and YWHAZ.

## Discussion

Brucellosis, caused by *Brucella*, is one of the common zoonotic diseases that poses a significant threat to global agriculture and livestock industries, as well as to human health, particularly among workers in the livestock sector [30]. In recent years, scientists have conducted detailed research on *Brucella* and identified 16 effector proteins [31]. These effector proteins play crucial roles in intracellular trafficking of *Brucella* and modulating host immune responses to promote virulence [32]. BtpA and BtpB are bacterial proteins containing domains homologous to the eukaryotic Toll/Interleukin-1 receptor (TIR) domain, initially identified in *Salmonella*, *Escherichia coli*, and *Brucella* [33, 34]. In eukaryotic immunity system, the TIR domain is critical for TLR signaling. It mediates protein interactions that activate Nuclear factor-kappaB (NF-κB) signaling pathway, leading to production of pro-inflammatory cytokines and type I interferons [35]. Bacterial TIR proteins are proposed to disrupt host immune responses through molecular mimicry of this signaling machinery, thereby inhibiting TLR-mediated defense pathways [36]. The *Brucella* effector protein BtpB inhibits the production of inflammatory cytokines in dendritic cells by interfering with the TLR-Myd88-MAL signaling pathway [37].

In order to maintain physiological homeostasis, cells undergo a form of programmed cell death known as apoptosis every day within the body [38]. During apoptosis, cells undergo morphological changes such as cell shrinkage, chromatin condensation, and DNA fragmentation, ultimately breaking down the dying cell into smaller fragments called apoptotic bodies, which are then phagocytosed by neighboring cells or phagocytes [39]. Many pathogenic bacteria rely on apoptosis to sustain persistent infections in the host. For example, when the intracellular parasite *Mycobacterium tuberculosis* infects the host cells, it can induce apoptosis in macrophages, thereby influencing the intracellular survival of the pathogen [40]. Research has shown that *Shigella flexneri* can prevent apoptosis by inhibiting effector caspases through LPS. The O-antigen moiety of LPS directly binds to caspases. Strains lacking the O-antigen or unable to replicate in the cytosol fail to block apoptosis and exhibit attenuated virulence [41]. *Brucella* can infect both professional and non-professional phagocytes. Although inhibiting host cell apoptosis likely confers a fitness advantage to the pathogen [42], no conclusive evidence has been established to confirm this hypothesis. Several *Brucella* virulence factors modulate host cell apoptosis, with both pro- and inhibitory effects reported. Current research has shown that Omp25 and Omp31 are the primary outer membrane proteins of *Brucella*, and together with BspJ, BspG and BspF, they function to inhibit apoptosis [43, 44]. In this study, we proved *Brucella* effect protein BtpB can promote apoptosis.

Accumulating evidence underscores the pivotal role of YWHAZ as a critical regulatory node in the cellular apoptosis network. Functioning primarily as a molecular scaffold and a regulator of 14-3-3 client protein sequestration, YWHAZ exerts a predominantly anti-apoptotic influence [45]. Growing evidence links its frequent upregulation to the regulation of cell growth and apoptosis [46]. The net effect is a significant enhancement of cellular resistance to diverse intrinsic and extrinsic apoptotic stimuli. YWHAZ depletion in liver cancer stem-like cells increases the expression of pro-apoptotic proteins Caspase 3 and Bax, while its overexpression in breast cancer cells reduces both proteins expression [47]. Neal CL et al*.* further reported that YWHAZ knockdown enhances cytochrome c release under low-serum conditions, sensitizing breast cancer cells to apoptosis by reducing procaspase−9 expression and caspase substrate cleavage [48]. Dysregulation of YWHAZ expression and function is frequently associated with pathological states characterized by apoptosis evasion, notably in cancer, highlighting its therapeutic potential. In this study, we have, for the first time, discovered that YWHAZ is involved in the regulation of cell apoptosis by the *Brucella* effector protein BtpB.

This study characterized the interactome of the *Brucella* effector protein BtpB and identified a physical interaction with the host regulatory protein YWHAZ through its TIR domain. We found that the decrease in YWHAZ protein is not attributable to transcriptional inhibition but rather results from degradation via the proteasomal and autophagic-lysosomal pathways. Recent studies have shown that the TIR domains of *Brucella* BtpA and BtpB possess intrinsic NAD⁺ hydrolase activity, leading to depletion of host cellular NAD⁺ levels during infection [14]. Given that NAD⁺ is a key regulator of cellular energy metabolism and survival signaling, its depletion alone can trigger metabolic stress and subsequently induce apoptosis. The NAD⁺ supplementation experiments in this study showed that treatment with the NAD⁺ precursors NMN partially rescued BtpB-induced apoptosis (Additional file 2). Consequently, we propose a dual model in which BtpB promotes apoptosis through both YWHAZ-dependent and NAD⁺ metabolism-dependent mechanisms. The relative contributions of each pathway warrant further investigation.

In summary, this study focused on the interaction between the *Brucella* effector protein BtpB and the host protein YWHAZ, elucidating the molecular details of how BtpB acts on YWHAZ. It confirmed that the effector protein BtpB is involved in regulating *Brucella*-induced cell apoptosis and investigated the impact of BtpB on apoptotic pathways. The study demonstrated that BtpB's regulation of cell apoptosis depends on its interaction with YWHAZ and revealed the influence of YWHAZ on the intracellular survival of *Brucella*. These findings have significant scientific implications for understanding the pathogenic mechanisms of *Brucella*, as well as for developing new vaccines and therapeutic targets against *Brucella*.

## Supplementary Information

**Additional file 1. Names and sizes of proteins interacting with BtpB**. Liquid chromatography-tandem mass spectrometry (LC-MS/MS) was employed to identify and systematically screen for host proteins that interact with BtpB.

**Additional file 2. NAD⁺ supplementation affects BtpB-mediated apoptosis.** In the NAD⁺ supplementation experiments, 1 mM of the NAD⁺ precursor NMN was added to the culture medium during the expression of BtpB and BtpBΔTIR, and a significant elevation of NAD⁺ levels was observed. It was shown that NAD⁺ levels in HeLa cells were significantly reduced by BtpB expression, whereas this effect was largely abolished by the BtpBΔTIR mutant, confirming that BtpB-mediated NAD⁺ consumption is dependent on its TIR domain. To investigate whether BtpB-induced apoptosis is affected by NAD⁺ supplementation, the protein levels of apoptosis-related genes were examined in cells with or without NMN treatment. It was found that the pro-apoptotic effect of BtpB was attenuated by NMN-mediated NAD⁺ elevation, suggesting that NAD⁺ metabolism is involved in BtpB-mediated apoptosis.

## Data Availability

The data presented in the current study are available from the corresponding author upon reasonable request. The data have been uploaded to the repository with the https://doi.org/10.6084/m9.figshare.31670521.
