# Supplementary material for: Effect of Brucella effector protein BtpB regulating apoptosis by targeting host protein YWHAZ
Source: Vet Res. 2026 Sep 24;57:171. doi: 10.1186/s13567-026-01840-9 (PMC13602538; doi:10.1186/s13567-026-01840-9)
Supplement: Supplementary file 1 [file 13567_2026_1840_MOESM1_ESM.docx]

**Additional file 1. Names and sizes of proteins interacting with BtpB**

| **Accession** | **Protein names** | **Gene names** | **MW [kDa]** |
| --- | --- | --- | --- |
| Q9NRN7 | L-aminoadipate-semialdehyde dehydrogenase-phosphopantetheinyl transferase | AASDHPPT | 35.75307083 |
| P11310 | Medium-chain specific acyl-CoA dehydrogenase, mitochondrial | ACADM | 46.55858475 |
| P68032 | Actin, alpha cardiac muscle 1 | ACTC1 | 41.99188264 |
| O95831 | Apoptosis-inducing factor 1, mitochondrial | AIFM1 | 66.85890999 |
| P54819 | Adenylate kinase 2, mitochondrial | AK2 | 26.46077324 |
| P04075 | Fructose-bisphosphate aldolase A | ALDOA | 39.3953077 |
| Q4VCS5 | Angiomotin | AMOT | 118.0126502 |
| P18085 | ADP-ribosylation factor 4 | ARF4 | 20.49769405 |
| Q9NZN5 | Rho guanine nucleotide exchange factor 12 | ARHGEF12 | 173.1251181 |
| P13637 | Sodium/potassium-transporting ATPase subunit alpha-3 | ATP1A3 | 111.6769167 |
| P24539 | ATP synthase F(0) complex subunit B1, mitochondrial | ATP5F1 | 28.89033694 |
| P36542 | ATP synthase subunit gamma, mitochondrial | ATP5F1C | 32.97527288 |
| O43684 | Mitotic checkpoint protein BUB3 | BUB3 | 37.13118113 |
| Q9NZT1 | Calmodulin-like protein 5 | CALML5 | 15.88277242 |
| P04040 | Catalase | CAT | 59.71875787 |
| Q86X02 | Cerebellar degeneration-related protein 2-like | CDR2L | 52.97800941 |
| P23528 | Cofilin-1 | CFL1 | 18.49065867 |
| Q96BP2 | Coiled-coil-helix-coiled-coil-helix domain-containing protein 1 | CHCHD1 | 13.46605018 |
| O75208 | Ubiquinone biosynthesis protein COQ9, mitochondrial | COQ9 | 35.4866083 |
| O75390 | Citrate synthase, mitochondrial | CS | 51.67951584 |
| Q13363 | C-terminal-binding protein 1 | CTBP1 | 47.50549733 |
| P00387 | NADH-cytochrome b5 reductase 3 | CYB5R3 | 34.21272416 |
| Q9H773 | dCTP pyrophosphatase 1 | DCTPP1 | 18.66936122 |
| P39656 | Dolichyl-diphosphooligosaccharide--protein glycosyltransferase 48 kDa subunit | DDOST | 50.76897233 |
| O15523 | ATP-dependent RNA helicase DDX3Y | DDX3Y | 73.10812461 |
| P31689 | DnaJ homolog subfamily A member 1 | DNAJA1 | 44.83945466 |
| Q13347 | Eukaryotic translation initiation factor 3 subunit I | EIF3I | 36.47861516 |
| Q9UBQ5 | Eukaryotic translation initiation factor 3 subunit K | EIF3K | 25.04344112 |
| Q9GZV4 | Eukaryotic translation initiation factor 5A-2 | EIF5A2 | 16.78223854 |
| Q15717 | ELAV-like protein 1 | ELAVL1 | 36.06915168 |
| Q9Y285 | Phenylalanine--tRNA ligase alpha subunit | FARSA | 57.52751244 |
| Q00688 | Peptidyl-prolyl cis-trans isomerase FKBP3 | FKBP3 | 25.16131813 |
| P20930 | Filaggrin | FLG | 434.9217077 |
| Q9HAV7 | GrpE protein homolog 1, mitochondrial | GRPEL1 | 24.2639196 |
| Q9Y2Q3 | Glutathione S-transferase kappa 1 | GSTK1 | 25.4802992 |
| P68871 | Hemoglobin subunit beta | HBB | 15.98829092 |
| Q9BXW7 | Haloacid dehalogenase-like hydrolase domain-containing 5 | HDHD5 | 46.29179434 |
| P06899 | Histone H2B type 1-J | HIST1H2BJ | 13.89557886 |
| P05114 | Non-histone chromosomal protein HMG-14 | HMGN1 | 10.65259384 |
| Q14103 | Heterogeneous nuclear ribonucleoprotein D0 | HNRNPD | 38.41030428 |
| P31942 | Heterogeneous nuclear ribonucleoprotein H3 | HNRNPH3 | 36.9030834 |
| Q6YN16 | Hydroxysteroid dehydrogenase-like protein 2 | HSDL2 | 45.36551796 |
| P61604 | 10 kDa heat shock protein, mitochondrial | HSPE1 | 10.92487173 |
| Q5T749 | Keratinocyte proline-rich protein | KPRP | 64.09324311 |
| P20700 | Lamin-B1 | LMNB1 | 66.3676257 |
| P40926 | Malate dehydrogenase, mitochondrial | MDH2 | 35.48073441 |
| Q9Y399 | 28S ribosomal protein S2, mitochondrial | MRPS2 | 33.22807848 |
| P82650 | 28S ribosomal protein S22, mitochondrial | MRPS22 | 41.25439797 |
| P13995 | Bifunctional methylenetetrahydrofolate dehydrogenase/cyclohydrolase, mitochondrial | MTHFD2 | 37.87119752 |
| P60660 | Myosin light polypeptide 6 | MYL6 | 16.91913397 |
| Q9GZZ1 | N-alpha-acetyltransferase 50 | NAA50 | 19.38603896 |
| P19338 | Nucleolin | NCL | 76.56836278 |
| P56556 | NADH dehydrogenase [ubiquinone] 1 alpha subcomplex subunit 6 | NDUFA6 | 17.85928813 |
| Q9BYG3 | MKI67 FHA domain-interacting nucleolar phosphoprotein | NIFK | 34.20103566 |
| Q15233 | Non-POU domain-containing octamer-binding protein | NONO | 54.19728369 |
| O43809 | Cleavage and polyadenylation specificity factor subunit 5 | NUDT21 | 26.21074089 |
| Q14980 | Nuclear mitotic apparatus protein 1 | NUMA1 | 238.115235 |
| P22061 | Protein-L-isoaspartate(D-aspartate) O-methyltransferase | PCMT1 | 24.62057247 |
| P30101 | Protein disulfide-isomerase A3 | PDIA3 | 56.74675008 |
| O43660 | Pleiotropic regulator 1 | PLRG1 | 57.15810441 |
| P13797 | Plastin-3 | PLS3 | 70.76621213 |
| P62937 | Peptidyl-prolyl cis-trans isomerase A | PPIA | 18.00088618 |
| O75475 | PC4 and SFRS1-interacting protein | PSIP1 | 60.06668258 |
| P25789 | Proteasome subunit alpha type-4 | PSMA4 | 29.46518138 |
| Q13200 | 26S proteasome non-ATPase regulatory subunit 2 | PSMD2 | 100.1357944 |
| P32322 | Pyrroline-5-carboxylate reductase 1, mitochondrial | PYCR1 | 33.33955828 |
| Q9UL25 | Ras-related protein Rab-21 | RAB21 | 24.33225664 |
| P63244 | Receptor of activated protein C kinase 1 | RACK1 | 35.0545595 |
| P62826 | GTP-binding nuclear protein Ran | RAN | 24.4076189 |
| Q96PK6 | RNA-binding protein 14 | RBM14 | 69.44890844 |
| Q9BWF3 | RNA-binding protein 4 | RBM4 | 40.28865027 |
| Q9BV68 | E3 ubiquitin-protein ligase RNF126 | RNF126 | 35.56228417 |
| P27635 | 60S ribosomal protein L10 | RPL10 | 24.58786878 |
| Q6NVV1 | Putative 60S ribosomal protein L13a protein RPL13AP3 | RPL13AP3 | 12.12692289 |
| P35268 | 60S ribosomal protein L22 | RPL22 | 14.77780478 |
| P62910 | 60S ribosomal protein L32 | RPL32 | 15.84980494 |
| P63173 | 60S ribosomal protein L38 | RPL38 | 8.212732425 |
| P62280 | 40S ribosomal protein S11 | RPS11 | 18.41899389 |
| P25398 | 40S ribosomal protein S12 | RPS12 | 14.50548231 |
| Q86WX3 | Active regulator of SIRT1 | RPS19BP1 | 15.42439588 |
| P60866 | 40S ribosomal protein S20 | RPS20 | 13.36430914 |
| P42677 | 40S ribosomal protein S27 | RPS27 | 9.454836185 |
| P08865 | 40S ribosomal protein SA | RPSA | 32.83343464 |
| P31151 | Protein S100-A7 | S100A7 | 11.46356499 |
| Q6UWP8 | Suprabasin | SBSN | 60.50459897 |
| P50454 | Serpin H1 | SERPINH1 | 46.41117943 |
| Q15637 | Splicing factor 1 | SF1 | 68.28590602 |
| Q15758 | Neutral amino acid transporter B(0) | SLC1A5 | 56.56225456 |
| Q00325 | Phosphate carrier protein, mitochondrial | SLC25A3 | 40.06873926 |
| O60264 | SWI/SNF-related matrix-associated actin-dependent regulator of chromatin subfamily A member 5 | SMARCA5 | 121.8283666 |
| P08621 | U1 small nuclear ribonucleoprotein 70 kDa | SNRNP70 | 51.52583605 |
| P09661 | U2 small nuclear ribonucleoprotein A' | SNRPA1 | 28.39813483 |
| P14678 | Small nuclear ribonucleoprotein-associated proteins B and B' | SNRPB | 24.59368831 |
| P62314 | Small nuclear ribonucleoprotein Sm D1 | SNRPD1 | 13.27336469 |
| Q5VX52 | Spermatogenesis-associated protein 1 | SPATA1 | 52.91298505 |
| Q08170 | Serine/arginine-rich splicing factor 4 | SRSF4 | 56.64533328 |
| Q16629 | Serine/arginine-rich splicing factor 7 | SRSF7 | 27.35002664 |
| Q9UNE7 | E3 ubiquitin-protein ligase CHIP | STUB1 | 34.83424977 |
| P53597 | Succinate--CoA ligase [ADP/GDP-forming] subunit alpha, mitochondrial | SUCLG1 | 36.2268468 |
| Q13148 | TAR DNA-binding protein 43 | TARDBP | 44.7113085 |
| Q13428 | Treacle protein | TCOF1 | 152.0153308 |
| O43615 | Mitochondrial import inner membrane translocase subunit TIM44 | TIMM44 | 51.32345694 |
| Q8N4H5 | Mitochondrial import receptor subunit TOM5 homolog | TOMM5 | 6.031371235 |
| P04637 | Cellular tumor antigen p53 | TP53 | 43.6253802 |
| P62995 | Transformer-2 protein homolog beta | TRA2B | 33.64556011 |
| Q9UI30 | Multifunctional methyltransferase subunit TRM112-like protein | TRMT112 | 14.19026037 |
| P68363 | Tubulin alpha-1B chain | TUBA1B | 50.11960685 |
| P55072 | Transitional endoplasmic reticulum ATPase | VCP | 89.26572387 |
| P63104 | 14-3-3 protein zeta/delta | YWHAZ | 27.72772965 |
